# Delayed innominate vein perforation and massive hemothorax following left-sided PICC placement: A case report

**DOI:** 10.1097/MD.0000000000050713

**Published:** 2026-09-11

**Authors:** Yong-Hwan Cho, Jin-Heon Jeong

**Affiliations:** a Department of Neurosurgery, Dong-A University Hospital, Busan, South Korea; b Department of Intensive Care Medicine and Neurology, Dong-A University Hospital, Busan, South Korea.

**Keywords:** Case report, Central venous catheterization, Chest radiography, Hemothorax, Peripherally inserted central catheter, Vein perforation

## Abstract

**Rationale:**

Peripherally inserted central catheters are common venous access devices; however, they can cause rare and life-threatening mechanical complications. Delayed vascular perforations often present with nonspecific findings, making early diagnosis challenging. Herein, we report a case of delayed innominate vein perforation, highlighting the importance of careful interpretation of serial chest radiographs.

**Patient concerns:**

An 82-year-old woman with acute ischemic stroke underwent bedside left-arm placement of a peripherally inserted central catheter. The initial postprocedural chest radiograph showed a catheter tip at the fifth thoracic vertebra with subtle proximal tortuosity; the tip appeared obliquely oriented relative to the expected central venous axis. The next day, the catheter tip had moved caudally, and the tortuosity resolved, which was misinterpreted as an improved position. Five days after the procedure, chest radiography revealed a massive right-sided pleural effusion.

**Diagnoses:**

Computed tomography confirmed that the delayed perforation of the innominate vein allowed the catheter to migrate into the mediastinum, resulting in a massive hemothorax.

**Interventions:**

The patient underwent urgent chest tube insertion followed by successful surgical repair of the perforated vein.

**Outcomes:**

The patient recovered without further complications and was discharged in a stable condition.

**Lessons:**

This case highlights a critical diagnostic paradox: the resolution of catheter tortuosity on follow-up chest radiography is normally a positive sign, indicating that the catheter tip had exited the vessel and released mechanical tension. Clinicians must interpret radiographic changes in catheter position with great caution and verify findings with functional assessments.

## 1. Introduction

Peripherally inserted central catheters (PICCs) are widely used for medium-to-long-term venous access. They are cost-effective, can be placed at the bedside, and have a lower risk of insertion-related mechanical complications compared to conventional central venous catheters.^[1]^ Although generally safe, PICCs are associated with risks, including catheter-related venous thrombosis, occlusion, and bloodstream infections.^[2–4]^ In addition, rare but life-threatening events, including delayed venous perforation and hemothorax due to catheter migration, have been reported.^[5]^

We present a case of delayed innominate vein perforation with massive hemothorax after left-sided PICC placement in an elderly patient. This case emphasizes the importance of cautious interpretation of postprocedural chest radiographs.

## 2. Case report

An 82-year-old woman with a history of diabetes mellitus, hypertension, and prior myocardial infarction presented with sudden onset of left-sided weakness and dysarthria. On arrival, motor power was Medical Research Council grade 4 in both the left upper and lower limbs, and her National Institutes of Health Stroke Scale score was 3. Brain magnetic resonance imaging revealed an acute infarction in the right basal ganglia. She was treated with dual antiplatelet therapy (aspirin and clopidogrel). She was admitted to the intensive care unit for management of acute ischemic stroke.

**Figure 1.**
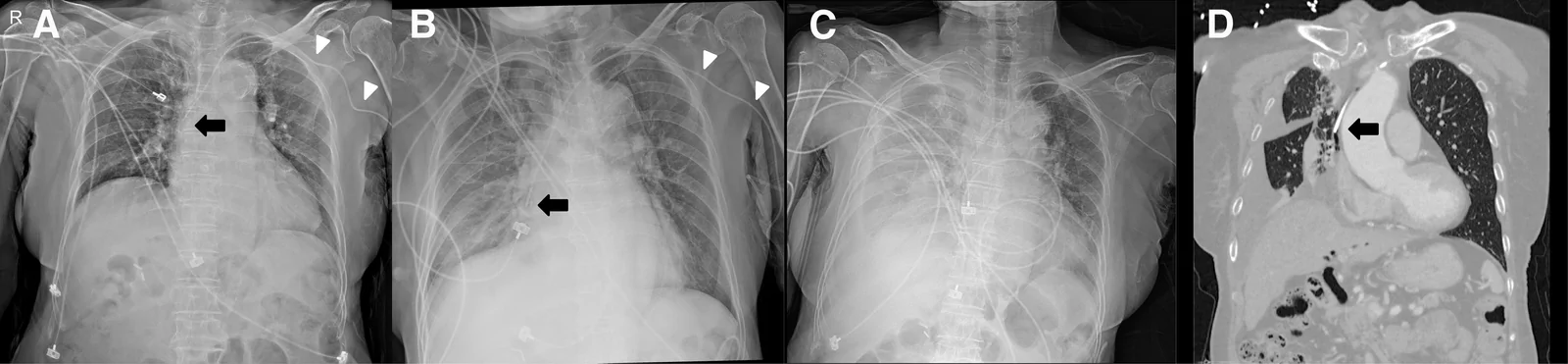
Serial chest radiographs and coronal CT image showing PICC progression and complications. (A) Chest radiograph taken immediately after peripheral central catheter insertion. The black arrow indicates the catheter tip at the midlevel of the fifth thoracic vertebra. White arrowheads mark the proximal portion of the catheter, which shows subtle tortuosity. The subtle tortuosity, considered together with the oblique orientation of the catheter tip relative to the expected central venous axis, was retrospectively interpreted as a potential sign of mechanical tension. (B) Chest radiograph obtained on postprocedural day 1. The catheter tip (black arrow) migrated caudally to the sixth thoracic vertebra. The previously observed tortuosity of the proximal catheter (white arrowheads) resolved, suggesting that this tension was released as the tip migrated caudally. (C) Chest radiograph on postprocedural day 5 demonstrating nearly total opacification of the right hemithorax, consistent with massive pleural effusion. (D) Coronal contrast-enhanced chest CT demonstrating extravascular migration of the catheter (black arrow) from the left innominate vein into the mediastinum. CT = computed tomography, PICC = peripherally inserted central catheter.

The following day, her neurological status deteriorated: motor power decreased to Medical Research Council grade 2 in the left upper limb and grade 3 in the left lower limb, and her National Institutes of Health Stroke Scale increased to 6. Induced hypertension therapy using intravenous phenylephrine was initiated, and a PICC was inserted via the left upper arm under ultrasound guidance at the bedside. The catheter was inserted smoothly without resistance during the procedure, and blood regurgitation confirmed intravascular placement. A postprocedural chest radiograph showed the catheter tip was located at the midlevel of the fifth thoracic vertebra, with subtle proximal tortuosity that was not considered abnormal at the time (Fig. 1A). During phenylephrine infusion, systolic blood pressure increased from 150 mm Hg to 180 mm Hg; however, neurological deficits did not improve.

On post-procedure day 1, chest radiography showed caudal migration of the catheter tip to the level of the sixth thoracic vertebra, with resolution of the previously noted tortuosity. This was interpreted as improved positioning in the superior vena cava rather than a sign of complication (Fig. 1B).

On post-procedure day 5, routine chest radiography revealed near-total opacification of the right lung field caused by massive pleural effusion, though the patient had no respiratory symptoms (Fig. 1C). Chest computed tomography demonstrated catheter migration into the mediastinum through a venous perforation (Fig. 1D). A chest tube was inserted, draining about 1700 mL of dark, hemorrhagic fluid. Broad-spectrum intravenous antibiotics (piperacillin-tazobactam) were initiated. Elective surgical repair via upper sternotomy was then performed, and the perforated innominate vein was successfully repaired. The patient recovered without further complications and was discharged in stable condition.

## 3. Discussion

PICCs are frequently used in critically ill patients due to their safety and ease of placement.^[1]^ However, rare but serious delayed complications, such as catheter migration and venous perforation, can occur.^[5]^ This case highlights the importance of careful interpretation of postprocedural chest radiographs. In our patient, catheter tip migration and resolution of tortuosity were misinterpreted as favorable signs, although they masked the development of a venous perforation (Fig. 1A–1C). This misinterpretation occurred because a 2-dimensional chest radiograph can obscure early signs of complications. Therefore, radiographic changes in the catheter position should be verified with functional assessments, such as checking for blood regurgitation, to avoid overlooking such complications.

The initial chest radiograph showed subtle proximal catheter tortuosity. Tortuosity on a single chest radiograph is a nonspecific finding and is not necessarily abnormal. In this patient, however, retrospective comparison of the serial radiographs showed that the tortuosity resolved in association with caudal migration of the catheter tip. The simultaneous straightening of the catheter and caudal migration of its tip suggested that mechanical tension within the catheter may have been released. Therefore, the initial tortuosity was retrospectively regarded as a potential sign of mechanical tension rather than an incidental finding. Even after uneventful insertion and confirmation of blood return, minor catheter advancement may occur during guidewire withdrawal owing to recoil or friction, potentially generating mechanical tension within the catheter.^[6]^ Subsequent arm movement can transmit force to the catheter and potentially cause gradual erosion of the vein wall.^[7]^ Thus, an apparent improvement in catheter configuration (straightening of prior tortuosity with tip migration) may paradoxically reflect release of mechanical tension and should prompt further evaluation rather than reassurance.

In our patient, the catheter inserted from the left arm did not appear to be aligned parallel to the expected axis of the superior vena cava on the initial chest radiograph. Left-sided PICC placement may carry an anatomical predisposition to central venous perforation because the catheter approaches the superior vena cava at a sharper angle, potentially increasing the likelihood of catheter tip impingement on the vessel wall.^[8]^ Persistent mechanical stress due to suboptimal orientation can lead to delayed perforation. Furthermore, the position of the catheter tip is dynamic; it can move approximately 2 cm with upper extremity movement, respiration, or changes in body position.^[7,8]^ This dynamic movement further increases the risk of vascular injury when the tip is already in contact with the vessel wall. In addition, the concurrent use of dual antiplatelet therapy may have aggravated the severity of the hemothorax once perforation occurred.

**Figure 2.**
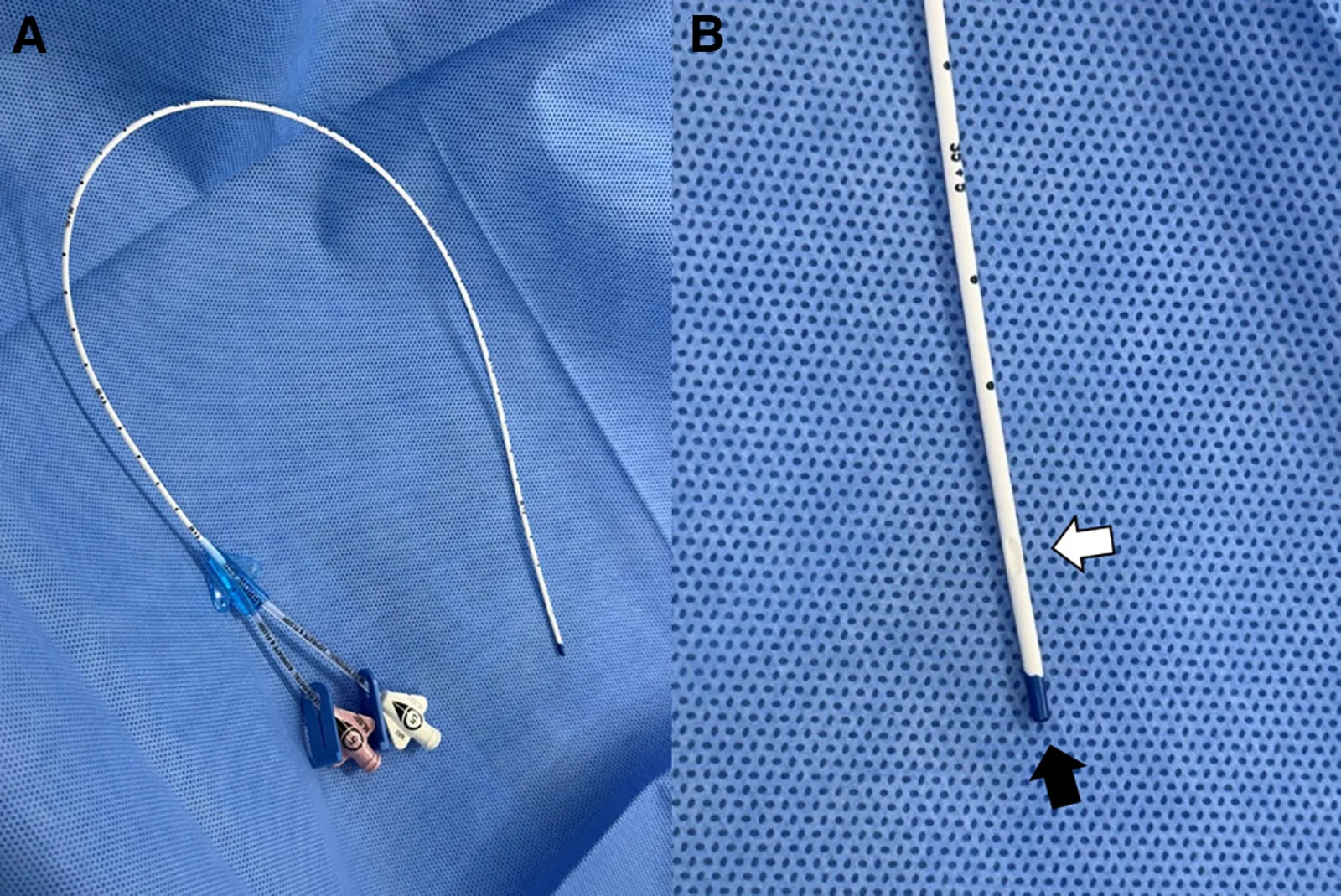
The PICC used in this case. The 5 French double-lumen polyurethane catheter (Arrow, Teleflex Inc.) features a distal tip with 1 end hole (black arrow) and one side hole (white arrow). The manufacturing process of the end hole resulted in a relatively stiff distal tip. PICC = peripherally inserted central catheter.

In this case, a 5 French double-lumen polyurethane PICC (Arrow, Teleflex Inc.) was used. Polyurethane provides the initial stiffness for insertion and then softens at body temperature, thereby improving intravascular tolerance.^[9]^ Notably, this catheter has a tip design with 1 end hole (black arrow) and 1 side hole (white arrow) (Fig. 2). The manufacturing process of the end hole makes the catheter tip relatively stiff. We hypothesized that this increased localized rigidity at the distal tip, combined with the suboptimal angle forcing it against the vessel wall, contributed to gradual endothelial injury and delayed perforation.

According to Pittiruti et al, late cardiac tamponade is virtually nonexistent with modern soft polyurethane catheters, and there is no evidence that positioning the catheter tip in the upper right atrium increases the risk of complications.^[10]^ We propose that this rigid tip, when chronically pressed against the innominate vein wall from a suboptimal angle, can cause a delayed perforation. This distinction is important, as the same group suggested that the “safe area” for tip placement includes the lower superior vena cava, cavoatrial junction, and upper right atrium. However, they also noted that left-sided catheters inserted too shallowly (leading to tip-to-wall contact) risk venous perforation, as seen in our case.^[10]^ Thus, to minimize the mechanical stress, the catheter tip should ideally be oriented parallel to the venous wall within a safe area. In addition, intracardiac electrocardiography can help verify the correct tip position by detecting the point of maximal *P*-wave positivity, which indicates the optimal location near the cavoatrial junction.^[11]^

Postprocedural radiographs should be evaluated not only for the catheter tip position, but also for subtle signs of mechanical stress, such as tortuosity and suboptimal tip orientation. If tension is suspected, withdrawing the catheter by 2 to 3 cm may relieve stress. However, if perforation is suspected to have already occurred, withdrawal can cause severe hemorrhage. In uncertain cases, functional assessment (e.g., blood regurgitation) and cross-sectional imaging are essential for safe clinical decision-making.

## 4. Conclusion

This case illustrates a rare but serious complication of left-sided PICC placement in which unrecognized catheter migration led to delayed innominate vein perforation and massive hemothorax. Serial follow-up chest radiographs should be carefully compared to prevent life-threatening outcomes, particularly when tortuosity is present.

## Author contributions

**Conceptualization:** Jin-Heon Jeong.

**Data curation:** Yong-Hwan Cho.

**Formal analysis:** Yong-Hwan Cho.

**Investigation:** Yong-Hwan Cho.

**Methodology:** Jin-Heon Jeong.

**Supervision:** Jin-Heon Jeong.

**Visualization:** Yong-Hwan Cho.

**Writing** – **original draft:** Yong-Hwan Cho.

**Writing** – **review & editing:** Jin-Heon Jeong.
